# Caring when the caregiver becomes ill: a qualitative analysis of the impacts and multidimensional challenges in informal caregiving

**DOI:** 10.15649/cuidarte.5416

**Published:** 2026-09-29

**Authors:** Inês Costa Durão, Sónia Guadalupe

**Affiliations:** 1 University of Coimbra, Faculty of Psychology and Educational Sciences and Faculty of Economics, Coimbra, Portugal. E-mail: inesdurao255@gmail.com University of Coimbra Coimbra Portugal inesdurao255@gmail.com; 2 University of Coimbra, Faculty of Psychology and Educational Sciences. Researcher at the Centre for Innovative Biomedicine and Biotechnology and the Centre for Health Studies and Research of the University of Coimbra, Portugal. E-mail: sonia.guadalupe@fpce.uc.pt University of Coimbra Coimbra Portugal sonia.guadalupe@fpce.uc.pt

**Keywords:** Informal Caregivers, Caregiver Burden, Social Support, Public Policies, Cuidadores Informales, Carga del Cuidador, Apoyo Social, Políticas Públicas, Cuidadores Informais, Sobrecarga do Cuidador, Apoio Social, Políticas Públicas

## Abstract

**Introduction:**

Informal care has enormous societal relevance in southern European countries. The illness of carers casts a shadow of doubt over the caregiving process.

**Objective:**

To analyze the multidimensional impacts and challenges when informal carers face a situation of illness or incapacity.

**Materials and Methods:**

A qualitative methodology was used, collecting data through semi-structured interviews and using categorical content analysis. The participating caregivers were recruited from the community through organizations.

**Results:**

10 informal carers participated, 9 women aged between 47 and 80 years old, who were family members and lived with the cared-for person. Two categories of analysis emerged: the impact on the informal carer and caring in a situation of illness or incapacity. The results showed impacts in the family, personal, social, work and financial life spheres, with restrictions on autonomy and freedom being highlighted. The carer's illness had implications for the provision of care, and there was a need to activate the informal support network, which was mainly family. There were also difficulties in accessing formal support.

**Discussion:**

Noteworthy the particularities of informal carers' experiences of illness, with increased difficulties compared to what other studies have shown in relation to informal care.

**Conclusion:**

The study contributes to highlighting the difficulties and needs of responding to and supporting carers who have fallen ill, posing challenges for social and health intervention in the community and for public policies.

## Resumen

**Introducción:**

El cuidado informal tiene una enorme relevancia social en los países del sur de Europa. La enfermedad de los cuidadores genera dudas sobre el proceso de cuidado.

**Objetivo:**

Analizar los impactos y desafíos multidimensionales cuando el cuidador informal se enfrenta a una situación de enfermedad o discapacidad.

**Materiales y métodos:**

Se utilizó una metodología cualitativa, con datos recopilados mediante entrevistas semiestructuradas y análisis de contenido categórico. Los cuidadores participantes fueron reclutados de la comunidad a través de organizaciones.

**Resultados:**

Participaron diez cuidadores informales, nueve de ellos mujeres, con edades comprendidas entre los 47 y los 80 años, familiares que convivían con la persona cuidada. El estudio analiza dos categorías: los impactos en el cuidador informal y el cuidado en una situación de enfermedad o discapacidad. Los resultados mostraron impactos en las esferas familiar, personal, social, laboral y económica, con una notable restricción de la autonomía y la libertad. La enfermedad del cuidador tuvo repercusiones en la prestación de cuidados, lo que requirió la activación de la red de apoyo informal, principalmente familiar. Se evidenciaron dificultades para acceder al apoyo formal.

**Discusión:**

Se destacan las particularidades de la experiencia de la enfermedad por parte de los cuidadores informales, evidenciando mayores dificultades en comparación con lo que muestran otros estudios sobre cuidados informales.

**Conclusiones:**

Este estudio contribuye a visibilizar las dificultades y las necesidades de respuesta y apoyo para los cuidadores que han enfermado, lo que plantea desafíos para las intervenciones sociales y sanitarias en la comunidad y para las políticas públicas.

## Resumo

**Introdução:**

O cuidado informal tem enorme relevância societária nos países do sul da Europa. A doença em cuidadores coloca um véu de dúvidas no processo de cuidar.

**Objetivo:**

Analisar os impactes e os desafios multidimensionais quando o cuidador informal enfrenta uma situação de doença ou de incapacidade.

**Materiais e métodos:**

Usou-se uma metodologia qualitativa com recolha de dados através de entrevistas semiestruturadas e com recurso à análise de conteúdo categorial. Os cuidadores participantes foram recrutados na comunidade através de organizações.

**Resultados:**

Participaram 10 cuidadores informais, 9 do sexo feminino, com idades entre os 47 e os 80 anos de idade, que eram familiares e coabitavam com a pessoa cuidada. O estudo analisa duas categorias: os impactes no cuidador informal e o cuidar numa situação de doença ou de incapacidade. Os resultados mostraram impactes nas esferas da vida familiar, pessoal, social, laboral, e financeira, sendo destacada a restrição na autonomia e na liberdade. A situação de doença do cuidador teve implicações na prestação de cuidados, tendo havido necessidade de ativar a rede de suporte informal, eminentemente familiar. Foram evidenciadas dificuldades de acesso ao apoio formal.

**Discussão:**

Destacam-se as particularidades da vivência da situação de doença por parte dos cuidadores informais, tendo sido potenciadas dificuldades acrescidas relativamente ao que outros estudos mostram em relação ao cuidado informal.

**Conclusões:**

O estudo contribui para evidenciar as dificuldades e as necessidades de resposta e de apoio aos cuidadores que adoeceram, colocando desafios à intervenção social e em saúde na comunidade e às políticas públicas.

## Introduction

Informal caregiving plays a crucial role in contemporary society, despite its social invisibility. The impact of caregiving on caregivers is experienced across multiple dimensions and becomes particularly challenging when informal caregivers themselves face health problems. Therefore, caregiving should be approached multidimensionally from a systemic perspective.

In Europe, it is estimated that 80% of all care is provided by informal caregivers, with approximately 44 million people (12% of the adult population) providing care informally, women being the most frequent caregivers^1^. Portugal has the highest rate of informal home care in Europe (12.4%), partly due to a shortage of workers in the formal care sector^2^. As the fourth most aged country in the world and the second most aged in Europe in 2024^3^, Portugal faces significant demographic challenges. The increase in longevity among the Portuguese population has not been accompanied by a corresponding increase in healthy life expectancy in old age. In 2022, life expectancy at age 65 reached 19.6 years, of which only 7.9 years were lived in good health^3^. Despite the heterogeneity of aging processes, older adults tend to require care due to age-related declines in functionality, illness, losses in their social networks, and increased vulnerability to loneliness, which may result in reduced autonomy or disability^4^-^6^.

Demographic trends and changes in family structure have led to an increasing number of people requiring care and, simultaneously, a decreasing number of available caregivers^5^,^7^, particularly due to the verticalization of intergenerational relationships^8^. The family system plays a central role in informal social support^4^, especially in providing care for dependent family members, reflecting the familistic nature of Portuguese society and its social protection system^8^.

The number of informal caregivers in Portugal is estimated to range between 800,000 and 1.4 million, with 25% providing full-time care^9^,^10^. Additionally, 98% of caregivers have a family relationship with the care recipient^11^. Informal caregivers are therefore predominantly family members and are typically women over 50 years of age^11^-^13^. Among OECD countries, approximately 60% of all daily informal care is provided by women, with Portugal ranking first in terms of gender imbalance, as 70.1% of informal caregivers are women^14^.

Informal caregivers are also more likely to experience physical health problems associated with both aging and caregiving, as well as mental health problems, when compared with non-caregivers of the same age^15^,^16^. However, literature has paid limited attention to this population, and there is a scarcity of knowledge regarding caregivers who themselves experience illness.

The process of assuming the caregiver role is dynamic and results from a combination of social, occupational, emotional, and economic factors^17^, as well as family ties, availability to provide care, proximity to the care recipient, perceptions regarding the caregiving role, and the ability to balance caregiving with personal and professional life^4^.

Informal caregiving is unpaid, but it is not always voluntary. It is often perceived both as a gift and as a moral obligation grounded in intergenerational reciprocity^18^. Within families and society, caregiving is frequently valued and associated with feelings of gratification, personal satisfaction, and personal growth^7^,^19^. Despite these positive aspects, informal caregiving can also have negative consequences across multiple dimensions, particularly in social, mental, and physical health, affecting both personal and professional life^7^,^19^, and increasing family conflict^7^.

Caregivers frequently report physical and emotional exhaustion, especially when caregiving extends over long periods. They commonly experience physical fatigue as well as symptoms of depression and anxiety^12^,^19^. Due to caregiver burden and reduced leisure time, informal caregivers also experience higher levels of isolation, stress, anxiety, depression, and low self-esteem, often neglecting their own health^20^.

It is important to highlight the implications of informal caregiving for caregivers’ professional lives, particularly given the challenges of reconciling paid employment with caregiving responsibilities^7^. This reveals a paradox in which exclusion from the labor market may result from an excessive workload across multiple spheres of life^9^. When caregivers cohabit with the care recipient, they are less likely to remain employed^12^. Even when adjustments are made to accommodate caregiving responsibilities—such as reduced working hours, flexible schedules, or temporary withdrawal from professional activities—caregivers’ professional performance and career progression^7^,^19^ may be adversely affected, further exacerbating gender inequalities. Studies focusing on informal caregivers who themselves experience illness remain scarce, reinforcing the relevance of this research in the Portuguese context.

Accordingly, this study aims to analyze the multidimensional impacts and challenges associated with caregiving when informal caregivers experience illness.

## Materials and Methods

**Type of study**

This was a qualitative study framed within an interpretive and socio-critical paradigm, aimed at recognizing and valuing participants’ subjective experiences. A descriptive cross-sectional design was adopted. This study followed the COREQ checklist (Consolidated Criteria for Reporting Qualitative Research). A non-probabilistic convenience sample was used, supported by strategic informants. The inclusion criteria were being the primary informal caregiver, being over 18 years of age, caring for a dependent person aged 65 years or older, and experiencing temporary unavailability to provide care due to illness or temporary disability.

Data was collected through semi-structured interviews. The interview guide was developed by the researchers based on the literature and included questions related to: (1) the context of informal caregiving and family organization; (2) personal experiences as an informal caregiver; (3) the impacts of providing informal care; (4) experiences related to illness and/or disability affecting the informal caregiver; and (5) current health status and perspectives regarding the future care of the dependent family member. These five broad dimensions were defined as a priori. Data analysis considered the meanings embedded in participants’ narratives according to the Grounded Theorizing framework^21^. Data triangulation was performed by two independent researchers, resulting in categories and subcategories consistent with the guiding dimensions. Theoretical saturation was achieved upon completion of the tenth interview.

**Procedures and ethics in research**

Participants were identified through community organizations in the Central Region of Portugal, based on the established inclusion criteria. Interviews were conducted between January and May 2024 after informed consent was obtained. The interviews were conducted by a researcher with a degree in Social Work. Each interview lasted approximately 30 minutes, was audio-recorded, and subsequently transcribed for analysis. Transcriptions preserved participants’ narratives in their original form, without grammatical correction, and were not returned to participants for review. Confidentiality during the interview process and data storage was ensured in accordance with the ethical standards of the Ethics Committee of the University of Coimbra, Portuguese data protection regulations, and the principles of the Declaration of Helsinki. The analysis process followed the criteria of credibility, reliability, and confirmability. The collected data are publicly available for consultation through Mendeley Data^22^ in the CareD database.

**Analysis**

The two researchers independently conducted a categorical content analysis of the semi-structured interviews. QDAMiner Lite software was used to support data organization and analysis. Five categories and their respective subcategories, along with their corresponding context units, were defined for the analytical process. The results related to two of these categories are presented in this article Figure 1.

**Figure 1:**
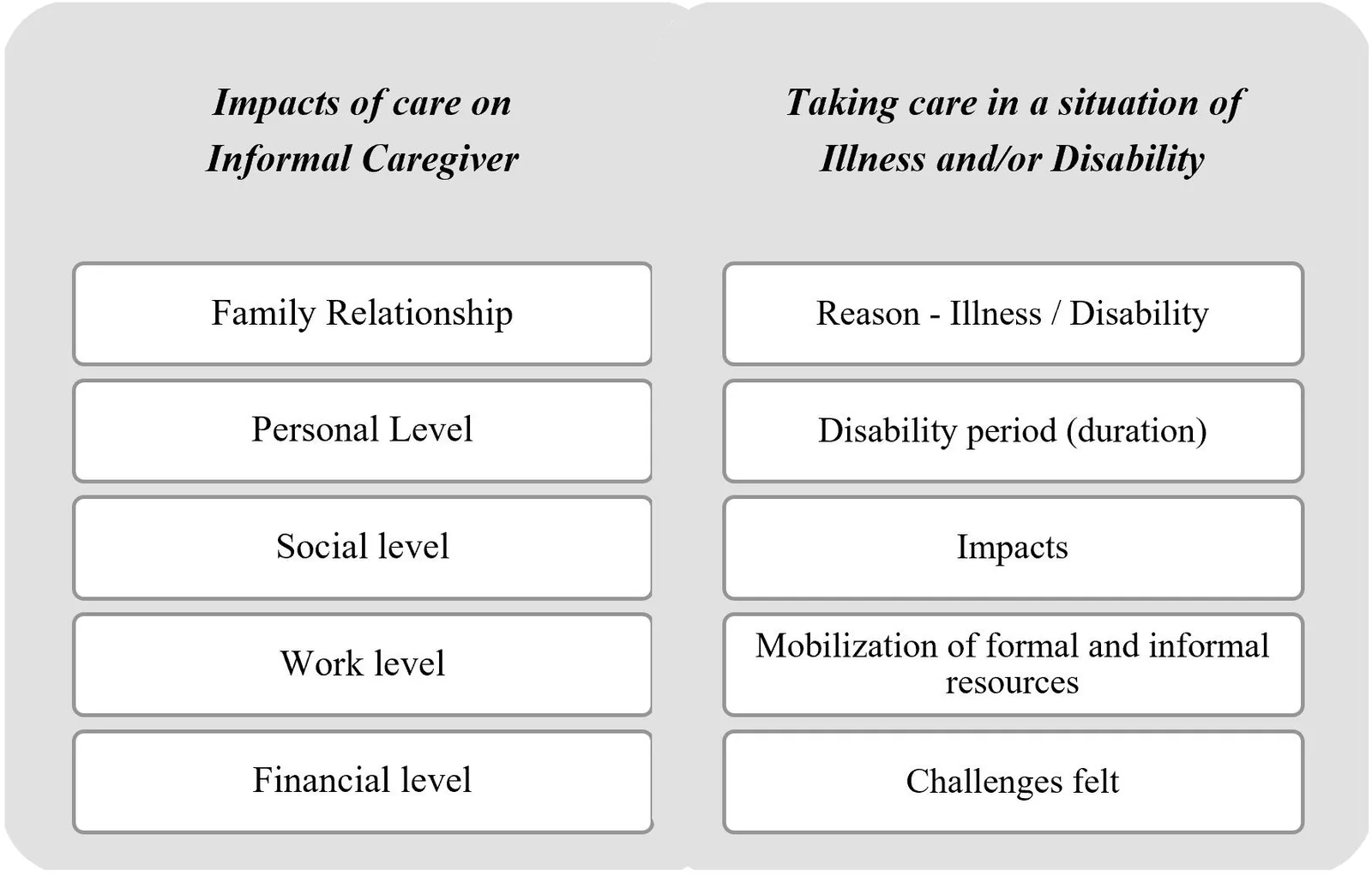
Conceptual map of data analysis

*Note: Figure 1 presents two categories of categorization obtained from the categorical analysis*

## Results

**Participants**

Ten informal caregivers participated in the study, predominantly women (80.00%) and married (80.00%), aged between 47 and 80 years (M = 59.10; SD = 9.80). Regarding employment status, 60.00% of participants were employed, while 40.00% were inactive, including three retired participants (30.00%) and one participant without paid employment (10.00%) Table 1. Participants were assigned identification codes from P1 to P10 according to the chronological order of the interviews.

**Table 1:** Profile of the Participating Informal Caregiver. n = 10

Code	Sex	Age (years)	Marital status	Situation regarding work	Relationship to the person being cared for
P1	Male	47	Married	Works for someone else	Mother-in-law
P2	Female	56	Married	Works for someone else, "Sick leave" (temporary incapacity for work)	Mother
P3	Female	56	Married	Works independently	Mother-in-law
P4	Female	57	Single	Without salaried work	Mother
P5	Male	73	Married	Retired	Wife
P6	Female	80	Married	Retired	Spouse
P7	Female	57	Married	Works for someone else	Mother
P8	Female	54	Married	Works for someone else	Mother
P9	Female	56	Widow	Retired due to disability	Father and Mother
P10	Female	55	Married	Works for someone else	Mother-in-law

P1 to P10 – Code assigned to each participant

To contextualize caregiving and the relationship between caregivers and care recipients, half of the participants provided care for a parent (50.00%), three cared for a mother-in-law (30.00%), and two cared for a spouse (20.00%). One participant provided care for two individuals. Most participants had siblings, except for P2, P6, and P7. In some cases, participants reported that their siblings had emigrated (P4 and P9). In 90.00% of cases, the care recipient lived in the same household as the caregiver, except for P3. In this case, the care recipient had temporarily moved into the caregiver’s home after becoming widowed, although they had previously lived as neighbors. Care was provided exclusively by 60.00% of participants (P2, P3, P4, P5, P6, and P9), whereas in 40.00% of cases (P1, P7, P8, and P10), caregiving responsibilities were shared with spouses, siblings, or other significant individuals.

The need for informal caregiving emerged following acute conditions in 40.00% of cases, while most situations resulted from chronic illnesses (60.00%). Across cases, participants reported increasing levels of dependency and growing care demands over time. Regarding caregiving duration, four participants had been providing care for more than 10 years (P4, P6, P7, and P8). The remaining participants had been providing care for less than 10 years, including one participant for less than one year (P2) and another for less than five years (P3).

In terms of time dedicated to caregiving, half of the participants devoted all their time to informal caregiving (P2, P4, P5, P6, and P9), with one participant stating, “This is my job, 24 hours a day” (P9). Other participants provided care at more specific times of the day, such as in the morning, at lunchtime, and/or at night. Participants P1 and P10 provided care during the morning and evening, complementing formal support provided by a day care center.

The results presented in this article focus on two categories: “Impacts of caregiving on the informal caregiver” and “Caregiving in situations of illness and/or disability.”

The category “Impacts of caregiving on the informal caregiver” highlights consequences at the family, personal, social, occupational, and financial levels Table 2.

**Table 2:** Multidimensional impacts on the informal caregiver. n = 10

Subcategory Impact / Unit of registration	% (n)
At the Family Level	
Family Dynamics	20.00 (2)
Family Relationships	20.00 (2)
No impact	50.00 (5)
Others	10.00 (1)
Difficulty managing routines and sleeping through the night	20.00 (2)
On a personal level*	
Restriction of autonomy to leave home or perform activities	70.00 (7)
Wear and tear or fatigue	60.00 (6)
Fear of possible recurrence of the disease	10.00 (1)
At a social level	
Restriction of freedom	80.00 (8)
No impact	20.00 (2)
At the Workplace level	
Reconciling work and caregiving (with adaptations)	50.00 (5)
Dismissal	10.00 (1)
No impact	40.00 (4)
At the financial level	
Increased expenses	50.00 (5)
Dependents cover expenses	20.00 (2)
No impact	20.00 (2)
Others	10.00 (1)

Note: *The variable allowed multiple responses. Therefore, the multiple responses for each variable justify the total percentage of responses being greater than 100%

When asked about family-level impacts, half of the caregivers reported no significant effects. However, regarding family dynamics, participants P1 and P7 indicated that caregiving had affected family relationships:

*“The only issue that perhaps stirred things up a bit is with the routines. Where are we going to take them, home, where are you going to pick them up from the day center, that dynamic until it's settled (...), until they're established, it also affects our own routine a bit, there's a slightly different dynamic, it involves many people, availability. It's one of the small challenges” (P1).*

*"I loved my grandchildren very much, I was always playing with them, and that's over now." (P7 about her mother who has been bedridden for 15 years).*

Strained family relationships were reported by participants P3 and P4, revealing that the lack of involvement of other family members in caregiving negatively affected family dynamics.

*“The only support I asked for was that they come by, talk to her, 'you see?' Ask questions, because if they did that, by doing so, they would realize the progression of the disease, which was what I fought so hard against, that it was getting worse” (P3).*

*“Of course we have problems with each other, because there are always things to do and it’s dealt with. Since I handle everything myself, it’s a bit complicated” (P4).*

Personal-level impacts were the most frequently reported across interviews.

Six caregivers described personal impacts related to fatigue and burnout:

*"It's just that whoever is taking care of someone like that, I think we get sick faster than the person themselves.", "And sometimes I say, may God never make me go through something like that again, because having to take care of someone like that is very painful" (P3).*

*“It’s a permanent, psychological exhaustion that then transforms into physical exhaustion, every day, every day, every day, every day, every day, every day” (P9).*

*“If I went back home, I couldn’t take another month like this (…) It wasn’t possible, I couldn’t take it like this anymore, because psychologically, I couldn’t take it anymore” (P10).*

Two participants indicated difficulty managing routines and difficulty sleeping at night:

*"Super complicated phase where she didn’t sleep a single night, she spent the night calling, calling all the children, and I basically spent the night here on the sofa. I wouldn’t wish this on anyone, on anyone at all” (P10).*

Finally, participant P1 expressed fear of relapsing into the condition that led to the addiction, stating:

*“In the early years, I felt, at least I, and she [my wife] also, some apprehension about the possible recurrence [of a stroke]” (P1).*

Regarding social impacts, participants P2 and P9 did not report significant effects in this domain. The remaining caregivers (80.00%) reported social impacts, as well as strategies used to cope with related challenges. Restrictions on freedom and autonomy were reported by eight participants, particularly in relation to leaving home and engaging in professional, leisure, or other daily activities.

*“Restrictions, constraints, limitations, because the simple fact that on a weekday, for example, we have the freedom to not have dinner at home today, to have dinner out, during that period of care we cannot do that. We have that schedule to follow, we must take care, yes” (P1).*

*“Sometimes we have activities that I belong to the community, and sometimes I can’t make it on Saturday mornings” (P7).*

*"That's how it is, we're more stuck at home" (P8).*

*“Of course, it takes away my autonomy. I’m more tied to her, I can’t go out, sometimes, a person is afraid she’ll do something stupid because it’s not the first time, she’s threatened (crying) (...) even downstairs when I go out, I must leave her a written note, I went to get bread, I went over there. And even then, sometimes, with that right in front of my eyes, it’s a problem. It raises problems in everything” (P5).*

*“I’m not going on vacation, I can’t, I have these tasks, we have these parts where we give up everything to always be there every day, either one or the other [sister]” (P8).*

*“We knew that during the month she was here you weren’t doing much, right? Like going downstairs for coffee. Someone had to stay with her like that” (P10).*

Several strategies were described by caregivers to cope with these limitations, including postponing scheduled events (P1), having one caregiver forgo social or professional commitments to remain with the care recipient (P1, P3, P7, and P10), and leaving the care recipient alone for short periods (P5 and P6), as illustrated in the following statements:

*“One day perhaps I would still be able to leave her here one day, perhaps, but no more” (P5).*

Other strategies mentioned include requesting someone to supervise the dependent person (P3, P4) and the use of video surveillance cameras (P1, P4):

*“I recently bought a video camera, which I use infrequently, because there isn’t much money to go out to dinner with friends or anything like that, and I wear an earpiece and listen to my mother. I watch or listen to see how she is doing, but it’s a rare occurrence” (P4).*

Regarding occupational impacts, some caregivers reported that caregiving had little or no effect on their professional lives, either because they were already on sick leave, retired, or receiving disability retirement benefits (P2, P5, P6, and P9).

Half of the caregivers reported being able to reconcile paid employment with informal caregiving responsibilities, either due to flexible work schedules (P3 and P7) or through various adaptations. These adaptations included advance planning of professional activities (P1), reduced working hours, changing from rotating to fixed shifts (P8), and requesting shift changes with colleagues (P10). One caregiver reported needing to leave work after her mother underwent surgery (P4).

Regarding the financial impacts, half of the caregivers report increased expenses, of which P1 and P2 indicate that the family can afford them:

*“That’s how it is, there are always expenses, although we usually say that the rice that feeds two, feeds three (...)”, “indirectly there were quite a few more expenses, but nothing exceptional that we couldn’t handle” (P1).*

*“I had more expenses because, look, the chair was borrowed, but I had to get her a walker and buy diapers. But thank God” (P2).*

Some caregivers report that the dependents themselves bear the expenses related to their care (P7 and P9), as one participant mentions:

*“No, it doesn’t affect me at all because my parents are financially independent. For now” (P9).*

Other participants reported no longer experiencing financial difficulties (P3 and P10). In contrast, P8 stated that although reduced working hours resulted in lower income, overall expenses were also reduced due to the decision not to use formal care services.

The category “Caregiving in situations of illness or disability” encompasses data related to the caregiving process when caregivers themselves experienced illness or disability. This category is divided into the following subcategories: (1) caregiver illness and/or disability; (2) implications for caregiving; (3) mobilization of formal and informal resources; (4) challenges faced; (5) caregivers’ current health status; and (6) perceptions regarding future caregiving.

Regarding the illness or disability experienced by caregivers, hospital-based treatments and surgical procedures were the most frequently reported situations. In most cases, the period of incapacity or temporary unavailability lasted several weeks or months.

In the case of P4, despite physical limitations following surgery, the participant continued to provide care immediately after the procedure. Several implications for caregiving were identified, including the need for rest, physical distance between the caregiver and the care recipient, and difficulties in performing caregiving tasks Table 3.

**Table 3:** Caregiver Illness and/or Disability, Period of Unavailability, and Implications for Caregiving. n = 10

Code	Caregiver's illness and/or disability	Period of unavailability (duration)	Implications
P1	Hernia	3-4 months of recovery.	The caregiver couldn't exert himself; he needed rest. "At that moment, he didn't have the availability to care for her, to hold Dona A's hand and take her here or there, I don't know, help her down a step or get into the car."
P2	Breast Cancer	Approximately 1 year of treatments.	When she undergoes oncology hospital treatments, she becomes unavailable; she mentions being tired. "Yes, tired, I was tired, but I treated her anyway."
P3	Covid-19	Unavailable for 1 week	Caregiver is unavailable to provide daily care.
P4	Sebaceous cyst surgery	Immediately after surgery.	The caregiver continued to provide care despite being prohibited from exerting herself; she reports feeling tired. “A person really needs to rest, which I didn’t do. I felt more tired, I had headaches, I was sleepier, but I did the work anyway. I forced myself to do it.”
P5	Hospital emergency due to loss of consciousness. Prostate cancer and heart problems	20 hours in emergency rooms and during cancer treatments.	During visits to the emergency room, she was unable to provide care, and when she underwent oncological hospital treatments, her availability also decreased significantly.
P6	Complex surgery. Caregiver with cancer problems	Two days a week with hospital treatments over several months.	When someone undergoes hospital treatment, they are no longer able to provide informal care. "In 2011, I spent more time in the hospital than almost at home."
P7	Surgery for a brain tumor	Hospitalization for 15 days and recovery at home.	The caregiver mentions the need for rest and notes that she experienced dizziness and sometimes couldn't see bright light, which made it difficult to perform caregiving tasks.
P8	Hand surgery	Sick leave for 1 month, but started taking care of things after 15 days.	The caregiver mentions the need for rest and increased difficulties in providing care during the first few weeks after surgery.
P9	Breast surgery. (previously: other surgeries and Covid-19)	1 week of hospital stay.	The caregiver mentions the need for rest and the inability to exert oneself during the initial recovery phase at home.
P10	Bowel surgery	Recovery over several weeks.	The caregiver mentions the need for rest. Due to the rotation of caregivers, after two months the dependent returned home. When questioned about the implications, she replies: "I think she had them all."

Regarding the mobilization of formal and informal resources in response to the caregiver’s illness or disability, 90.00% of participants reported strengthening support from their existing informal network (except for P4). New requests for support were also identified within the informal network (P3, P6, and P7) and the formal network (P2) Table 4.

Regarding caregiving support networks, all participants mentioned at least one person who provides support in caregiving or someone they could rely on if needed, whether through formal or informal support. For 60.00% of the sample, the caregiving support network was mixed. It was exclusively informal in 30.00% of cases and exclusively formal in 10.00%. The formal support network mobilized included home support services (P3, P7), day centers (P1, P10), formal in-home caregivers (P3, P4), physiotherapy services (P6, P9), and parish council services for activities for older adults (P9).

**Table 4:** Mobilization of Formal and Informal Support Resources During the Caregiver's Illness or Disability Period. n = 10

Subcategory	Appealed % (n)	Did not appeal % (n)
Strengthening the support of the existing informal network	90.00 (9)	10.00 (1)
New requests for support on the informal network	30.00 (3)	70.00 (7)
New support requests on the formal network	10.00 (1)	90.00 (9)

Table 5 details the mobilization of formal and informal support, including excerpts from interviews illustrating how participants managed the impact of the caregiver’s illness on the caregiving process for the dependent person. Most participants (80.00%) reported barriers to accessing formal support services. These barriers included high financial costs (P2, P6, P8), difficulties related to daily logistics, a preference for receiving care at home from an informal caregiver (P4, P7, P9), and concerns about the perceived quality of services (P5, P8, P9).

**Table 5:** Mobilization of Formal and Informal Resources in the context of Illness or Disability of the Informal Caregiver. n = 10

Code	Mobilization of formal and informal resources
P1	Strengthening the informal support network. The participant stated that he did not feel the need to seek additional support, although his wife experienced an increased caregiving burden. As he explained. “In the weeks when she wasn’t there, I could rest, and in the weeks when she was, I burdened Mrs. C a little more; she was the one who did everything”. The participant also mentioned the temporary availability of support from neighbors.
P2	Strengthening the informal support network and a new request for support from the formal network. The participant reported increased support from her husband and cousin. She also mentioned that, when she was unable to stay with her mother, she asked a neighbor to care for her through a paid service. Home care services were suggested by the hospital; however, the caregiver declined this option, explaining: “They said they could only come once a week to clean the house, cook, and take care of her. Once a week wasn’t going to solve anything, so I gave up”.
P3	Strengthening the informal support network and a new request for support within the informal network. The caregiver temporarily stopped providing care. During the first few days, caregiving responsibilities were assumed by her husband, followed by a brother-in-law. However, due to his limited availability because of work commitments, the family decided to leave the dependent person in the care of the mother-in-law of one of the brothers-in-law. As the participant explained, “For me, COVID was the opportunity I had to finally step away, allowing them to stay with her and realize the condition she was in”.
P4	There was no strengthening of the support network or any new request for formal or informal support.
P5	Strengthening the informal support network. During the caregiver’s treatment, he stayed with his wife in a residential care facility. He reported having requested assistance from staff members while he was in the emergency room. During that emergency episode, his daughter stayed with her parents for a few days to provide additional support. As he explained, “Yes, when I was in the emergency room for 20 hours, I asked the staff at the residence to keep an eye on her and help her become familiar with the room, because even after 8, 10, or 15 days there, she still wasn’t fully comfortable with the door.”
P6	Strengthening the informal support network and a new request for informal support. During the caregiver’s hospital treatment, the couple’s daughter assumed caregiving responsibilities, and the dependent person stayed overnight at her home. When the daughter was unavailable, another woman stayed overnight at the couple’s home to provide care for the dependent person. As the participant explained, “Sometimes my daughter would come, especially when she was on vacation. Otherwise, a woman from Póvoa de Sobrinhos would come and stay overnight at my house.”
P7	Strengthening the informal support network and a new request for informal support. During the recovery period, due to the caregiver’s temporary incapacity, her sister assumed caregiving responsibilities. At times, the sister also sought assistance from a cousin to help with caregiving.
P8	Strengthening the informal support network. During her recovery, her sister provided care.
P9	Strengthening the informal support network. During the surgery and recovery period, the caregiver was no longer able to provide care and required care herself. Her sister, daughter, and mother provided support to both the caregiver and the dependent person. As the participant explained, “At first, my daughter was here; she even washed my clothes and shirts, visited me, and brought me everything I needed. Then she left, and my sister came. If I hadn’t had them, well, I don’t know (laughs).”
P10	Strengthening the informal support network. During her recovery, the caregiver received increased support from family members and neighbors in providing care for her mother-in-law.

Regarding the challenges experienced, 50.00% of participants reported difficulties related to the caregiver’s own recovery, while 30.00% identified the impossibility of leaving the dependent person alone. Participants also reported feelings of isolation during this process, the burden associated with caregiving, and challenges related to caregiver support services.

*“There were times when I felt somewhat unsupported by the social services, that is, while, yes ma'am, I was at the day center, but in fact, when you are limited in your functions it seems like there's no one else, let's say, you need a little help, there's no one” (P1).*

*“I think all caregivers should have a psychologist, that’s what I think, so they can talk about things they don’t talk about with anyone else, because people don’t even understand, because they don’t go through the same thing, they don’t know what it’s like to be caregivers” (P4).*

*"I think theywereverybigchallenges, psychologicalones, I hadalotofpatience, itwasverycomplicated" (P10).*

Regarding the caregiver’s current health status, half of the participants reported feeling well (P3, P4, P6, P8, and P10). The remaining participants reported health problems and limitations related to their illness or recovery process. Regarding perceptions of the future of caregiving, most participants anticipated a worsening of the dependency situation (40.00%). Others expressed concern about the future (20.00%), while some believed they would continue providing care as long as they remained healthy (30.00%). In contrast, P4 and P6 reported not thinking about the future. In the case of P10, the family member receiving care had died (10.00%).

*“If I’m healthy and ready, I’ll see it through to the end. If not, something must be sorted out later, right?” (P2).*

*“I don’t know what’s going to happen, or when, I can’t be thinking about the future, at this moment thinking about how I’m going to take care of things from now on, what’s going to happen, because I don’t know. Challenges arise and we try to solve them” (P4).*

## Discussion

The profile of the informal caregivers participating in this study was predominantly female, with a mean age of approximately 60 years. Most were married and employed. All participants provided care to family members (mother, father, mother-in-law, or spouse) and lived with the care recipient. This profile is consistent with that identified in studies conducted in Portugal^11^,^13^,^23^,^24^.

The results revealed that caregiving had multidimensional impacts on the lives of informal caregivers, affecting family, personal, social, occupational, and financial domains. At the family level, half of the participants reported no significant impact. When impacts were identified, these were related to family routines and dynamics, as well as relationships between family members, with increased conflict associated with caregiving responsibilities. At the personal level, all caregivers reported some type of impact, including restricted autonomy to leave home or engage in personal activities, burnout, fatigue, difficulties managing daily routines, and sleep disturbances.

At the social level, caregivers reported restrictions on their own freedom and that of their family members, particularly regarding leisure activities and vacations. At the occupational level, employed caregivers reported adjusting balance paid work and caregiving responsibilities, including greater flexibility, reduced working hours, reduced professional activities, or self-employment. One participant left her job to care for her mother because she was unable to reconcile caregiving with her professional responsibilities, while another was temporarily unable to work. Finally, in financial terms, half of the participants reported increased household expenses associated with providing informal care.

The literature also highlights the multidimensional nature of these impacts. Families experience changes in their routines when they begin providing informal care^13^. Family conflicts have also been reported, particularly among siblings^7^. In the personal domain, several impacts on caregivers have been identified^7^,^18^,^19^. Notably, the loss of freedom to leave home and engage in personal activities may contribute to social isolation^15^. Being an informal caregiver has also been associated with reduced leisure time, reflecting the burden experienced by caregivers^25^,^26^.

Balancing work and caregiving responsibilities presents significant challenges and may lead to job abandonment, particularly when caregiving is prolonged^27^,^28^. In line with the present findings, a study conducted in Portugal found that 52.50% of caregivers were active in the labor market and reported no significant impact on their working lives. However, some participants reported difficulties related to justifying absences and sick leave, reduced working hours, income-related problems, challenges in balancing professional and family responsibilities, burnout^11^. Support from social protection systems for informal caregivers remains limited, even after the formal recognition of caregiver status in Portugal, particularly when caregiving is associated with increased financial burden^27^.

This study also examined informal caregiving in situations where caregivers experienced illness or disability, focusing on the impacts and challenges faced, the formal and informal support mobilized, and future perspectives regarding caregiving.

The illness of the informal caregiver represents a significant challenge, with clear repercussions for the caregiving process. Participants highlighted issues such as physical distance between the caregiver and the care recipient, inability or difficulty in performing caregiving tasks, fatigue, and an increased need for rest. Half of the caregivers identified their own recovery process as a major challenge, followed by difficulties related to not being able to leave the care recipient alone, the burden associated with caregiving, challenges in accessing support services, and feelings of isolation throughout the caregiving process. These impacts and challenges reveal the limited availability of resources to help caregivers cope with the accidental crisis caused by illness, highlighting the need for greater support. In this context, the mobilization of both formal and informal resources becomes essential. In response to caregiver illness, 90.00% of participants strengthened their informal support network, 30.00% sought additional resources within the informal network, and 10.00% sought support from the formal network. A clear preference for informal social support was evident.

The literature suggests that caregivers are likely to experience high levels of stress when they lack the internal or external resources necessary to cope with crises such as illness, particularly when pre-existing health problems are present^29^. The importance of mobilizing additional resources, including support from other caregivers, has also been highlighted as a strategy to mitigate some of the impacts and challenges identified in this study^29^.

When asked about their perceptions of the future of caregiving for their family members, participants highlighted the anticipated worsening of the dependency situation, followed by the belief that caregiving would continue as long as the caregiver remained healthy. Concerns about the future, as well as avoidance of thinking about it, were also reported.

Analysis of participants’ narratives revealed feelings of hope, uncertainty, and sadness, findings that are also consistent with those reported by Guadalupe et al^27^. The future may present additional challenges for informal caregiving, both due to the worsening health condition of the dependent family member and the deterioration of the caregiver’s own health. Evidence suggests that caregivers are themselves at risk of illness, and that this risk may be reduced through stronger formal support systems for caregiving^30^.

This study has limitations that should be acknowledged. First, difficulties were encountered in sample recruitment due to the specificity of the target population, despite the use of non-probabilistic sampling techniques. In addition, some analytical categories would benefit from greater saturation, particularly those related to occupational and personal impacts. The study adopted a homogeneity criterion regarding caregivers’ experiences of temporary disability and clinical inability to provide care, excluding situations involving mental health conditions. Future research should include a broader range of illness and disability situations among caregivers, with particular attention to the implications of the duration of incapacity for caregiving and the degree of temporary disability experienced by the caregiver. Studies with larger samples and quantitative, correlational designs are recommended to explore potential associations between variables.

## Conclusions

The findings highlight that informal caregiving has multidimensional impacts on both caregivers and their families. The personal, social, and occupational impacts are particularly significant. At the personal and social levels, notable challenges include restrictions on autonomy to leave home, reduced leisure time, and experiences of fatigue and burnout. At the occupational level, employed participants reported being able to balance caregiving and work through adjustments to their professional activities and work schedules, although in some cases informal caregiving led to temporary or permanent withdrawal from employment.

Caregiver illness and/or disability further intensifies the impacts and challenges associated with caregiving, revealing an increased need for social support. The study also identified barriers to accessing formal social services and to combining formal and informal sources of support.

Given the barriers to accessing formal support, greater monitoring and follow-up by primary health care and community social services are essential, particularly in situations involving caregiver illness, as caregivers often feel isolated and unsupported throughout the caregiving process. Social Work play a crucial role in supporting this population by safeguarding rights and well-being, strengthening social support networks, and mitigating the adverse impacts of informal caregiving.

What happens when the informal caregiver becomes ill? This fundamental question motivated the present study. Given the scarcity of studies addressing this specific situation in scientific literature, this research represents a relevant contribution to the existing body of knowledge and provides a solid foundation for future studies in this field.

Informal caregivers play a vital social role. However, they are also vulnerable to illness or disability that may compromise their ability to provide care. When faced with such circumstances, where can they turn and on whom can they rely? The findings clearly indicate that caregivers primarily rely on their informal social support networks, particularly family ties, highlighting the family-centered nature of caregiving in Portugal^8^.

Participants’ narratives also suggest that maintaining informal caregiving remains a central priority, if caregivers are physically able to assume caregiving responsibilities and remain in good health.

This study highlights the need for integrated interventions combining health care and community-based social care to ensure effective support for informal caregivers. Strengthening public policies aimed at supporting caregivers is essential. In particular, the implementation of integrated support programs for informal caregivers is recommended, with special attention to caregivers at risk of illness or already experiencing illness. Additionally, community-based social care services for dependent populations should be re-evaluated and made more flexible to better respond to caregiving needs.
